# Cytomegalovirus Reactivation in Patients With Acute Lymphoblastic Leukemia in Non-transplant Setting

**DOI:** 10.7759/cureus.32936

**Published:** 2022-12-25

**Authors:** Mansoor Abbas, Sameen Bin Naeem, Mussadique Ali Jhatial, Syed W Bokhari, Bushra Ahsan, Usman Ahmad

**Affiliations:** 1 Medical Oncology, Shaukat Khanum Memorial Cancer Hospital and Research Centre, Lahore, PAK

**Keywords:** cmv viremia, immunosuppression, cytomegalovirus reactivation, adolescent and young adults, acute lymphoblastic leukemia

## Abstract

Acute lymphoblastic leukemia is predominately a childhood disease and around two third of cases are of B-cell phenotype. Cytomegalovirus is an important cause of morbidity and mortality in allogeneic hematopoietic progenitor cell transplant; however, it is rare in patients with B-cell acute lymphoblastic leukemia in non-transplant settings. In this study, we evaluated 72 patients of acute precursor (pre) B-cell acute lymphoblastic leukemia at Shaukat Khanum Memorial Cancer Hospital and Research Center, out of which three were positive for Cytomegalovirus.

## Introduction

Acute lymphoblastic leukemia (ALL) is predominantly a disease of childhood and around two third of the cases are of B-cell phenotype (B-cell ALL) [1,2]. Age is one of the prognostic factors for B-cell ALL with older age associated with a poorer prognosis [3]. B-cell ALL in adolescents and young adults (AYAs) fairs less well regarding survival outcomes as compared to children mainly because of higher incidence of unfavorable cytogenetics and comorbid medical conditions leading to poor tolerability of intensive pediatric inspired protocols [4].

Around 83% of people are seropositive for Cytomegalovirus (CMV) in general population, with CMV infection acquired in the first three years of life [5]. Once acquired in childhood, CMV infection remains latent in later years of life. CMV reactivation is an important cause of morbidity and mortality in allogeneic hematopoietic progenitor cell transplant (HPCT) settings [6]. However, it is relatively rarer (15-26%) in patients with B-cell ALL in a non-transplant setting [7,8]. Among those in non-transplant settings, CMV reactivation and disease manifestation is rarer in teenage and young adult patients as compared to the pediatric age group [9,10]. We are presenting a study of 72 patients treated at our center, out of which three went on to develop CMV reactivation during treatment - two patients early on during induction and the third during maintenance phase.

## Materials and methods

**Figure 1:**
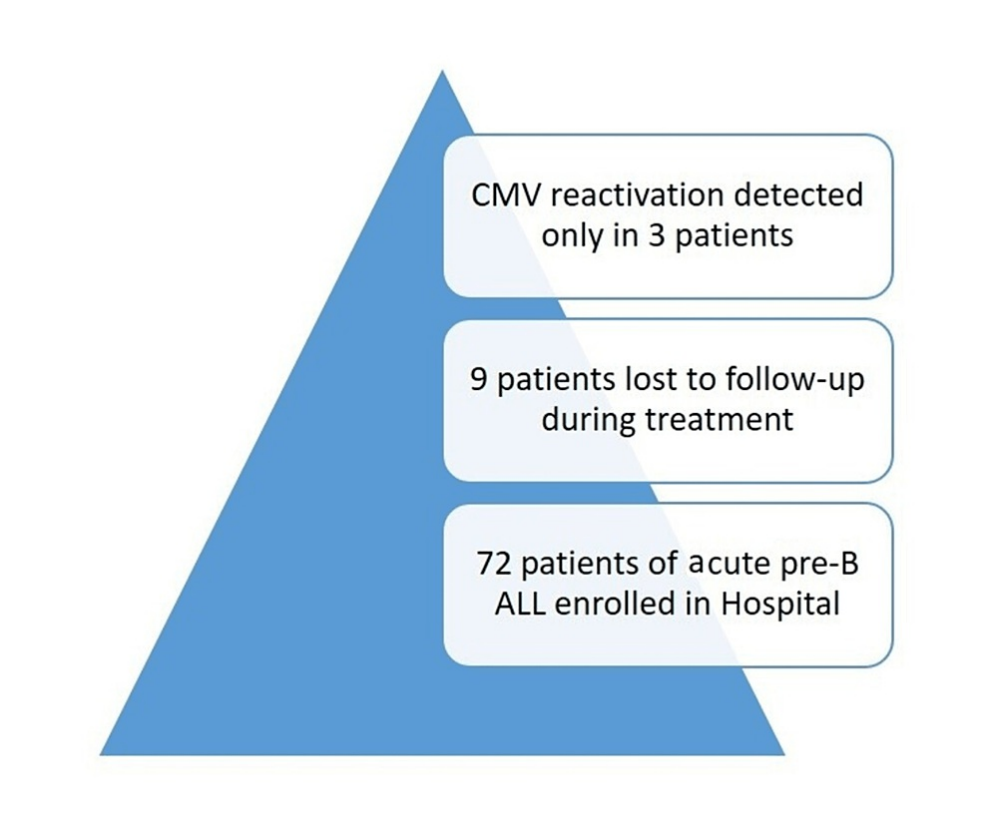
Number of patients enrolled in the hospital, out of 72 patients, only three were detected with CMV reactivation. CMV: cytomegalovirus; ALL: acute lymphoblastic leukemia; pre: precursor

A retrospective study was done using the Hospital Information System (HIS) of Shaukat Khanum Memorial Cancer Hospital for collection of data. Data were extracted for 72 patients of ALL treated in the last three years after getting institutional board review approval (approval number: EX 30-11-22-01). Patients who developed CMV reactivation during their treatment course for ALL in the non-transplant setting were identified. CMV viremia was defined as >10,000 copies/mL of CMV DNA using polymerase chain reaction (PCR). Only three patients were identified to be positive for CMV infection (Figure 1).

## Results

Baseline patient and disease characteristics

Out of 72 patients registered in our hospital with a diagnosis of precursor (pre) B-cell ALL, only 63 were treated. Rest of nine patients lost to follow-up during their course of treatment. The median age of patients was 21 years with a range of 19-53 years. Fifty-one patients were male (81%) and 12 were female (19%). All the above patients had no significant personal history of any comorbid conditions or any exposure to chemicals or radiation in past. Out of these 63 patients, three patients had a family history of breast cancer, one each with colorectal and hepatocellular carcinoma. Normal cytogenetics was reported in 22 patients (35%), and abnormal cytogenetics was reported in 41 patients (65%). All patients were negative for mixed lineage leukemia (MLL) gene rearrangements. However, breakpoint cluster region-Abelson 1 (BCR/ABL) translocation was positive in two patients (3%). Post-induction day 29, measurable residual disease (MRD) was positive in 28 patients (44%). Out of these 63 patients, 31 patients (49%) relapsed and 31 patients are still alive at their last follow-up visit (Table 1).

**Table 1:** Demographic details and disease characteristics of patients enrolled in hospital. MRD: measurable residual disease; CMV: Cytomegalovirus; MLL mixed lineage leukemia; BCR/ABL: breakpoint cluster region-Abelson 1

Patient and disease baseline characteristics		Count
Gender	Female	12
	Male	51
Personal history	Nil	63
Family history	Breast cancer	3
	Colorectal carcinoma	1
	Hepatocellular carcinoma	1
	Not significant	58
Chemical exposure	Nil	63
Cytogenetics	Normal male karyotype	16
	Abnormal male karyotype	36
	Normal female karyotype	6
	Abnormal female karyotype	5
MLL status	Positive	0
	Negative	63
BCR/ABL status	Positive	2
	Negative	61
Day 29 MRD status	Positive	28
	Negative	35
Relapse	Yes	31
	No	32
CMV status	Negative	60
	Positive	3
Status at last visit	Alive	31
	Dead	32

Patients with CMV reactivation

Case 1

A 21-year-old male with precursor B-cell acute lymphoblastic leukemia (pre B-cell ALL) who had already received almost one year of Berlin-Frankfurt-Munster chemotherapy protocol (daunorubicin, vincristine, PEG-asparaginase, cyclophosphamide, cytarabine, intrathecal methotrexate, mercaptopurine) and seven months of maintenance (vincristine, oral mercaptopurine, and oral methotrexate), reported to have on and off fevers for three months. He presented in the emergency department a few times with fever, diarrhea, and malaise managed with hydration and paracetamol. Each time he was discharged after work-up on supportive medications and empirical antibiotics. He was represented to the emergency department with high-grade fevers and pain in right lumbar region. The blood cultures requested during previous emergency department visits were negative. Complete blood count was significant for low absolute lymphocyte count with a normal absolute neutrophil count (ANC). C-reactive protein (CRP) was high. Liver and renal function tests were within normal limits. Malarial parasite film was negative, antibodies for Brucella were negative, and blood, urine, and stool cultures did not show any growth. Polymerase chain reaction for coronavirus disease 2019 (COVID-19) was negative. PCR for CMV DNA showed a very high titer of 93,800 copies/mL. Patient was commenced on ganciclovir 5 mg/kg (350 mg) IV every 12 hours. His fever settled after three days of commencing on ganciclovir. He was discharged with instructions to continue intravenous ganciclovir for total two weeks and then switch to oral valganciclovir to be continued till two weeks after CMV PCR is negative. He achieved a complete response (CR), and maintenance chemotherapy was restarted which was held during CMV treatment.

Case 2

An 18-year-old female with pre B-cell ALL received Berlin-Frankfurt-Munster 2000 induction chemotherapy (daunorubicin, vincristine, PEG-asparaginase, prednisolone, intrathecal methotrexate). She was in the cytopenic phase after completion of induction chemotherapy with negative MRD when she developed fever and presented to the emergency room. She was neutropenic and lymphopenia with an ANC and absolute lymphocyte count of 0.58x10^3^ and 0.66x10^3^, respectively, and a platelet count of 42,000 thousand. She was admitted and commenced on IV antibiotics and supportive measures. However, after three days of empirical broad-spectrum antibiotics and antifungals, she was still spiking fevers and had developed a generalized maculopapular rash. PCR for CMV and Ebstein bar virus was sent, and she was commenced on acyclovir. However, she deteriorated with ongoing fever spikes, shortness of breath, and got intubated for type II respiratory failure. CMV PCR came back positive with 93,000 copies/mL. She was commenced on ganciclovir 5 mg/kg IV every 12 hours. She had raised inflammatory markers and triglyceride levels. A bone marrow biopsy was done to rule out hemophagocytic lymphohistiocytosis (HLH). She fulfilled the criteria for HLH and was treated along the lines of HLH secondary to CMV infection, with steroids and IV etoposide. She improved gradually and was extubated and eventually discharged from the hospital. She resumed her consolidation and was in complete remission from leukemia perspective. However, in the subsequent months, she had a persistently positive minimal residual disease (MRD) and was given blinatumomab. This resulted in complete response, and she is currently being prepared for allogeneic stem cell transplant.

Case 3

A 19-year-old male with pre B-cell ALL was admitted for BFM 2000 induction protocol (daunorubicin, vincristine, PEG-asparaginase, prednisolone, intrathecal methotrexate). His inpatient stay was complicated by transaminitis, hyperbilirubinemia, and fever. He was receiving empirical piperacillin/tazobactam and fluconazole, which was switched to imipenem due to deranged liver functions and growth of *Escherichia coli* on urine culture, sensitive to carbapenems. Amphotericin B was added later for continued fever spikes. CT scan of chest showed extensive bilateral ground glass opacities. He continued to deteriorate and was shifted to the intensive care unit because of his respiratory distress. His clinical condition kept on worsening, and he continued to have fever spikes. *Mycobacterium tuberculosis* GeneXpert and PCR for *Pneumocystis jirovecii* were negative. Bronchoalveolar lavage (BAL) culture grew *Aspergillus fumigatus* for which he was treated. He was stepped down to inpatient floor after his respiratory parameters improved; however, he continued to spike fevers. His PCR for CMV returned positive for 10,700 copies of DNA per milliliter and was consequently commenced on intravenous ganciclovir 5 mg/kg every 12 hours with improvement in liver functions and fever. He got better in a few days and remained fever-free. He was deemed unfit for BFM 2000 consolidation and was started on methotrexate/mercaptopurine maintenance. Unfortunately, his leukemia subsequently relapsed (Table 2 suggests baseline patient and disease characteristics positive for CMV).

**Table 2:** Demographics and disease characteristics of patients positive for CMV reactivation. MRD: measurable residual disease; CMV: Cytomegalovirus; MLL mixed lineage leukemia; BCR/ABL: breakpoint cluster region-Abelson 1

Variables	Case 1	Case 2	Case 3
Age at presentation (years)	21	18	19
Gender	Male	Female	Male
Co-morbid conditions	None	None	None
Marital status	Single	Single	Single
Radiation exposure	None	None	None
Chemical exposure	None	None	None
Previous chemotherapy exposure	None	None	None
Genetic conditions	None	None	None
Family history of cancer	None	None	None
Hemoglobin (g/dL)	10.2 (12-15)	10 (12-15)	5.1 (12-15)
Platelets (10^3^/µL)	22,000 (150-450)	1,38,000 (150-450)	1,56,000 (150-450)
White cell counts (10^3^/µL)	13 (4-10)	7.49 (4-10)	2.04 (4-10)
Bone marrow review	70% blasts	8% blasts	30% blasts
Cytogenetics	Complex karyotype	Complex karyotype	Complex karyotype
MLL gene rearrangement	Not detected	Not detected	Not detected
BCR/ABL translocation	Not detected	Not detected	Not detected
MRD status at baseline	87% Blasts	3% Blasts	8% Blasts
MRD status post-induction therapy on day 29	Negative	Positive	Negative

## Discussion

CMV is a betaherpesvirus and is almost universally carried by human population throughout the world [11]. The seropositive for CMV infection is around 83% and prevalence is directly related to age [12]. Primary infection is usually asymptomatic in individuals with competent immune systems, usually acquired in childhood but infection is never eliminated, and a life-long latent infection is established [13]. While the humoral and innate immune responses play a role in the early phase of infection, cellular immunity is required to control its latency, prevent reactivation, and inhibit progression to overt infection. Immunocompromised states secondary to human immunodeficiency virus/acquired immunodeficiency syndrome (HIV/AIDS) or immunosuppression in patients with solid organ or hematopoietic stem cell transplant results in reactivation of CMV. CMV disease is a major cause of death in stem cell and organ transplant recipients and people with HIV/AIDS.

Our patients did not undergo stem cell transplantation and did not have HIV/AIDS. Data on adult patients with cancer and leukemia who have not undergone transplantation are scanty. All three patients were febrile and had low lymphocyte counts. One of them developed CMV pneumonia, second patient had CMV-related hemophagocytic lymphohistiocytosis (HLH), and the third one had hepatic involvement with CMV (Table 3).

**Table 3:** Laboratory values at the time of CMV reactivation. INR: international normalized ratio; LDH: lactate dehydrogenase; CMV: Cytomegalovirus

Lab parameter	Case 1	Case 2	Case 3	Normal parameter
White blood cells	0.13	0.78	2.72	4-10 (x10^3^/µL)
Lymphocytes	0.24	0.68	0.75	1-5 (x10^3^/µL)
Hemoglobin	10.8	8.3	8.7	12-15 (g/dL)
Thrombocytes	157	33	70	150-450 (x10^3^/µL)
Prothrombin time	9.6 s	14.3	12.2	9-14 (s)
INR	1.09	1.36	1.13	
Fibrinogen	215	97.1	83.8	200-400 (mg/dL)
LDH	135	442	542	135-214 (U/L)
Ferritin	60	2,312	N/A	5-148 (ng/mL)
Triglycerides	115	1,108	168	<150 (mg/dL)
Cytomegalovirus DNA copies/mL	93,800	90,800	10,700	No copies
C-reactive protein	22.9	276	14	<5 (mg/L)

Treatment with ganciclovir resulted in negative viral titers in all three patients. Two of the three patients went on to relapse, one is lost to follow-up. Diagnosis of CMV reactivation can be elusive as it is a rare entity with non-specific signs and symptoms. The index of suspicion needs to be high to catch it early on in the course so that it can be treated timely.

This study had a few limitations. This is a retrospective study and we were unable to establish a true causality. There are no consensus guidelines to check CMV status in non-transplant acute pre B-cell acute lymphoblastic leukemia patients as established in post-allogeneic stem cell transplanted patients of pre B-cell acute lymphoblastic leukemia. However, the risk of CMV reactivation among patients is higher with lymphopenia during their course of treatment. However, this also cannot be concluded whether its due to disease-related or CMV reactivation itself is the causative factor of lymphopenia. Further research is needed in order to establish the guidelines to check CMV reactivation status in non-transplant settings.

## Conclusions

CMV reactivation is very rare in teenage and young adults pre B-cell ALL in non-transplant and non-HIV settings. The number of cases is too low to draw any conclusions about associations; however, a low lymphocyte count seems to be a common link. All three patients not only had an aggressive disease course but two of those patients had poor tolerability to chemotherapy. Nevertheless, CMV reactivation should be in the differential diagnoses in acute lymphoblastic leukemia patients with fever, especially if the lymphocyte counts are low. Additionally, as it is common practice in patients undergoing allogeneic stem cell transplant, pre-emptive therapy with antivirals and regular monitoring of CMV PCR cannot be recommended with the current data.
